# Identifying Three Ecological Chemotypes of *Xanthium strumarium* Glandular Trichomes Using a Combined NMR and LC-MS Method

**DOI:** 10.1371/journal.pone.0076621

**Published:** 2013-10-02

**Authors:** Fangfang Chen, Fuhua Hao, Changfu Li, Junbo Gou, Dayan Lu, Fujun Gong, Huiru Tang, Yansheng Zhang

**Affiliations:** 1 CAS Key Laboratory of Plant Germplasm Enhancement and Specialty Agriculture, Wuhan Botanical Garden, Chinese Academy of Sciences, Wuhan, China; 2 CAS Key Laboratory of Magnetic Resonance in Biological Systems, State Key Laboratory of Magnetic Resonance and Atomic and Molecular Physics, Wuhan Centre for Magnetic Resonance, Wuhan Institute of Physics and Mathematics, Chinese Academy of Sciences, Wuhan, China; 3 Graduate University of Chinese Academy of Sciences, Beijing, China; Imperial College London, United Kingdom

## Abstract

Xanthanolides, as the sesquiterpene lactones, are reportedly the major components for the pharmacological properties of *X. strumarium* L. species. Phytochemical studies indicated that the glandular structures on the surface of plant tissues would form the primary sites for the accumulation of this class of the compounds. As the interface between plants and their natural enemies, glandular trichomes may vary with respect to which of their chemicals are sequestered against different herbivores in different ecologies. However, to date, no data are available on the chemical characterisation of *X. strumarium* glandular cells. In this study, the trichome secretions of the *X. strumarium* species originating from nineteen unique areas across eleven provinces in China, were analysed by HPLC, LC-ESI-MS and NMR. For the first time three distinct chemotypes of *X. strumarium* glandular trichomes were discovered along with the qualitative and quantitative evaluations of their presence of xanthanolides; these were designated glandular cell Types I, II, and III, respectively. The main xanthanolides in Type I cells were 8-epi-xanthatin and xanthumin while no xanthatin was detected. Xanthatin, 8-epi-xanthatin, and xanthumin dominated in Type II cells with comparable levels of each being present. For Type III cells, significantly higher concentrations of 8-epi-xanthatin or xanthinosin (relative to xanthatin) were detected with xanthinosin only being observed in this type. Further research will focus on understanding the ecological and molecular mechanism causing these chemotype differences in *X. strumarium* glandular structures.

## Introduction

*X. strumarium* L. is an annual herb that belongs to the Asteraceae family [1]. The plant is traditionally used for the treatment of rhinitis, rheumatism, tuberculosis, cancer, ulcers and malaria [2-5]. Due to its multiple bioactivities especially anti-tumor and anti-cancer activities [6,7], this plant has attracted much scientific interests. Most of its pharmacological effects are attributed to the presence of sesquiterpene lactones called xanthanolides [8-10]. Two xanthanolide sesquiterpene lactones, 8-epi-xanthatin and 8-epi-xanthantin-5β-epoxide, showed significant inhibitions of the proliferation of several human tumour cells including A549, SK-OV-3, SK-MEL-2, XF498, and HCT-15 *in vitro* [11]. Moreover, the xanthanolides have been considered as a promising drug against methicillin-resistant *Staphylococcus aureus* [12]. Despite their pharmaceutical importance, the biosynthesis of the xanthanolide sesquiterpene lactones in *X. strumarium* remains largely unknown. To understand their specialised metabolism, it is essential to know which tissue or specific structures in the plants are the primary sites for biosynthesising the targeted compounds. We have found that xanthanolide sesquiterpenes were highly biosynthesised at early leaf stage and accumulated in the glandular cells on the surfaces of the *X. strumarium* tissues. The novel multi-cellular glandular structure consisting of 6-pairs of cells was firstly isolated from *X. strumarium* plants. As the interface for interactions between plants and environmental factors such as pests and microbes, the glandular cells might be differently and genetically evolved for the accumulation of specialised metabolites responsive to unique ecological regions. For example, two chemotypes of glandular trichomes have been found in the anti-malarial plant *Artemisia annua* in an evolutionary context [13,14]. *X. strumarium* is widely distributed in China and has long been used as a herbal medicine for many years [15]. However, little is known about the chemical variations of *X. strumarium* glandular trichomes in response to different ecological areas.

**Figure 1:**
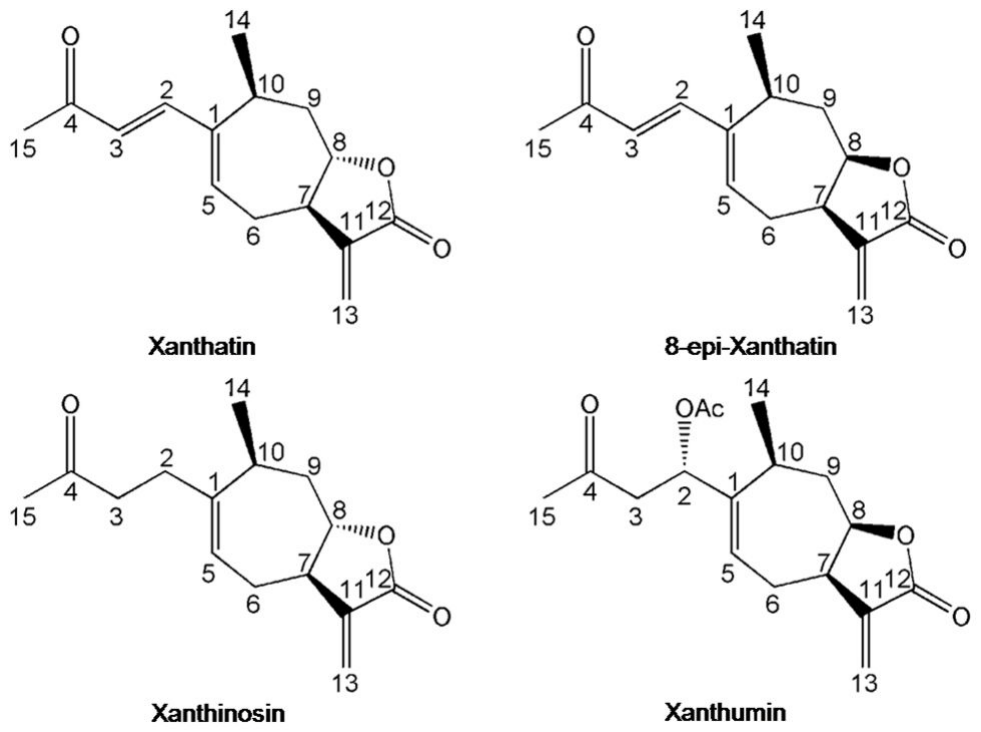
Structures of some xanthanolides in the *X. strumarium* species analyzed in this study.

Therefore, we investigated the composition of xanthanolide sesquiterpene lactones (Figure 1) in *X. strumarium* glandular trichomes from nineteen unique ecological areas of eleven provinces in China with the combined LC-MS and NMR techniques. Our objectives in this study were (1) to find whether or not different chemotypes of the glandular structure were present for *X. strumarium* species as in the case of the anti-malarial plant *A. annua* and (2) to characterise these chemotypes in terms of their relative abundance of the major xanthanolide sesquiterpene lactones.

## Materials and Methods

### Chemicals and plant materials

Methanol-d_4_ (99.8%) was obtained from Cambridge Isotopes Laboratories (Andover, MA). All other solvents used in this study were HPLC grade. Xanthatin standard was purchased from BioBioPha Company (Yunnan, China). All the *X. strumarium* plant materials were identified by Prof. Jianqiang Li at Wuhan Botanical Garden, Chinese Academy of Sciences. The plant seeds were collected from eleven provinces in PR China including Hubei (Xianning, Wuhan, Xishui and Fangxian), Anhui (Langxi and Hefei), Hunan (Huaihua), Jiangxi (Ganzhou), Zhejiang (Lishui), Sichuan (Suining, Guanyuan and Qingcheng), Gansu (Qiangyang), Henan (Sanmenxia and Nanyang), Guizhou (Wangmo and Zunyi), Shandong (Taian) and Beijing (Figure S1). The seeds were germinated and cultivated in the greenhouse of Wuhan Botanical Garden, Chinese Academy of Sciences. For the leaf materials used in this study, the first and second leaves from the top were considered to represent the young leaves and the remainders were deemed to be mature. None of the locations or activities mentioned in this study required for specific permissions. We also confirmed that the field studies did not involved in endangered or protected species.

### Scanning electron microscopy (SEM) analysis and the glandular trichome isolations

For the SEM analysis and glandular trichomes isolations, the plant materials of the *X. strumarium* species from Hubei-Wuhan were used. The surface of the plant materials including young leaves, mature leaves, stems, flowers, and seed coats was examined under Environmental Scanning Electron Microscope (Quanta 200, FEI Co. Ltd, The Netherlands) at an accelerating voltage of 15 kV under low vacuum. To isolate the glandular trichomes, the protocol described previously by Covello et al. was applied with some modifications [16], the young leaves were abraded in a cell disrupter by glass beads in an isolation buffer (25 mM MOPSO, pH 6.6, 200 mM sorbitol, 10 mM sucrose, 5 mM thiourea, 2 mM dithiothreitol, 5 mM MgCl_2_, 0.5 mM sodium phosphate, 0.6% (w/v) methylcellulose and 1% (w/v) polyvinylpyrrolidone (Mr 40000)). The disrupted extracts were filtered through 425 µm nylon mesh, and the filtrate was then successively passed through a 350, 250, 125, 100, 75, 58 and 42 µm square aperture nylon meshes. The isolated glandular trichomes were retained on the 42 µm mesh.

### Chemical extraction and purification

To investigate xanthanolide biosynthesis in different organs, fresh plant materials from young leaves, mature leaves, stems, flowers, and seeds were powdered and extracted with chloroform. The chloroform extracts were evaporated under reduced pressure and re-dissolved in methanol for HPLC and LC-ESI-MS analysis. For purifying the targeted compounds, about 100 g of intact young leaves were collected and immersed in 2 L of chloroform and extracted by consecutive sonication for 30 min in an ice bath. The chloroform extracts were concentrated under vacuum and re-suspended in 2 mL of methanol for purification by preparative HPLC. An Eclipse-C18 semi-prep column (9.4 mm×250 mm, 5 µm) was used at 25 °C with an injection volume of 100 µL, using a UV detector. The mobile phase consisted of methanol (A) and water (B) containing 0.1% (v/v) formic acid in a stepped gradient mode as follows: 0 to 40 min, 40 to 50% A. The flow rate was 4 mL/min. The monitoring wavelength was set to 280 nm and the peak fractions of the targeted compounds were collected followed with drying under vacuum.

### HPLC and LC-MS analysis

HPLC analysis was performed on a Shimadzu LC-20AT HPLC system using an Inertsil ODS-SP reverse phase column (5 µm, 250 mm × 4.6 mm) (Shimadzu, Kyoto, Japan). The mobile phase consisted of acetonitrile (A) and water (B) containing 0.1% (v/v) formic acid in a stepped gradient mode as follows: 0 to 20 min, 70 to 40% A; 20 to 30 min, 40% A; 30 to 30.1 min, 40 to 70% A; 30.1 to 40 min, 30% A. The flow rate was 0.8 mL/min and the column oven temperature was 25 °C, and a photodiode array detector was used. The monitoring wavelength was set from 190 to 800 nm.

For LC-ESI-MS analysis, samples were separated on an Accela max 600 HPLC system equipped with an online degasser, a quaternary solvent delivery system, an autosampler, a UV detector and a TSQ Quantum Access Max (Thermo Scientific, USA) with an electrospray ionisation (ESI) source. An analytical *Agilent* C18 column (5 µm, 250× 4.6 mm) was used with a flow rate of 0.8 mL/min and a column oven temperature of 25 °C. The mobile phases consisted of water (A) and acetonitrile (B) both containing 0.1% (v/v) formic acid in a stepped gradient mode which was the same as that previously described. The monitoring wavelength was set to 280 nm. A T-piece was used as a splitter so that analytes from the analytical columns were detected by the UV detector and MS system in parallel with only about 20% elution directed to the mass spectrometer. The acquisition of the mass spectra was conducted in positive ion mode with a sheath gas of pressure 40 bar, an auxiliary gas pressure of 10 bar, a vaporiser temperature of 250 °C, a capillary temperature of 350 °C, and a capillary offset of 35. The spray voltage was set to +3000 V. The MS data were recorded with ranges of m/z 50–400.

### NMR spectroscopy experiments

For NMR analysis, purified samples (1 to 2 mg) were transferred into a microtube (2 mL) with addition of 800 µL *methanol-d*_*4*_, then vortexed at high speed to expedite dissolution. Following centrifugation (16,000 g at 4 °C for 10 min), 600 µL of supernatant for each sample was transferred to 5 mm NMR tubes for analysis.

### Identification of compounds by NMR analysis

The full assignments of the proton and carbon signals of these xanthanolide sesquiterpene lactones (Figure 1) were based on ^1^H NMR and 2D NMR spectra from 600 and 800 MHz spectrometers and other published data [17-19]. Five 2D NMR spectra were recorded and processed as previously reported [20-22] for *X. strumarium* glandular trichomes extracts including ^1^H-^1^H TOCSY, ^1^H-^1^H COSY, JRES and ^1^H-^13^C HSQC and ^1^H-^13^C HMBC.

For assignment purposes, one 1D ^1^H spectrum and five 2D spectra were recorded at 298 K on a Bruker AV III 600 NMR spectrometer equipped with a 5 mm BBO probe operating at 600.13 MHz and a Bruker AV 800 MHz NMR spectrometer equipped with a 5 mm TCI CyroProbe operating at 800.20 MHz (Bruker Biospin, Germany), respectively. For the 1D ^1^H NMR spectrum, the NOESYPR1D sequence was used. Residual water suppression was achieved with a weak irradiation during the recycle delay (RD, 2 s) and mixing time (tm, 80 ms for the Bruker 600 MHz and 100 ms for the Bruker 800 MHz). A total of 16 transients were collected into 32 k data points for the Bruker 600 MHz and 64 k data points for the Bruker 800 MHz, for each spectrum with a spectral width of 20 ppm, and an acquisition time of 1.36 s for the Bruker 600 MHz and 2.04 s for the Bruker 800 MHz. An exponential window function with a line broadening factor of 1 Hz was applied to all free induction decays (FIDs) before Fourier transformation (FT). Based on the method described previously [21,23], the relative abundances of the metabolites were evaluated from the integrals of selected metabolite NMR signals (least overlapping signals) relative to that of 8-epi-xanthatin (We set it as 1).

## Results and Discussion

### Xanthanolide biosynthesis was associated with the distribution of glandular cells on *X. strumarium* organs

**Figure 2:**
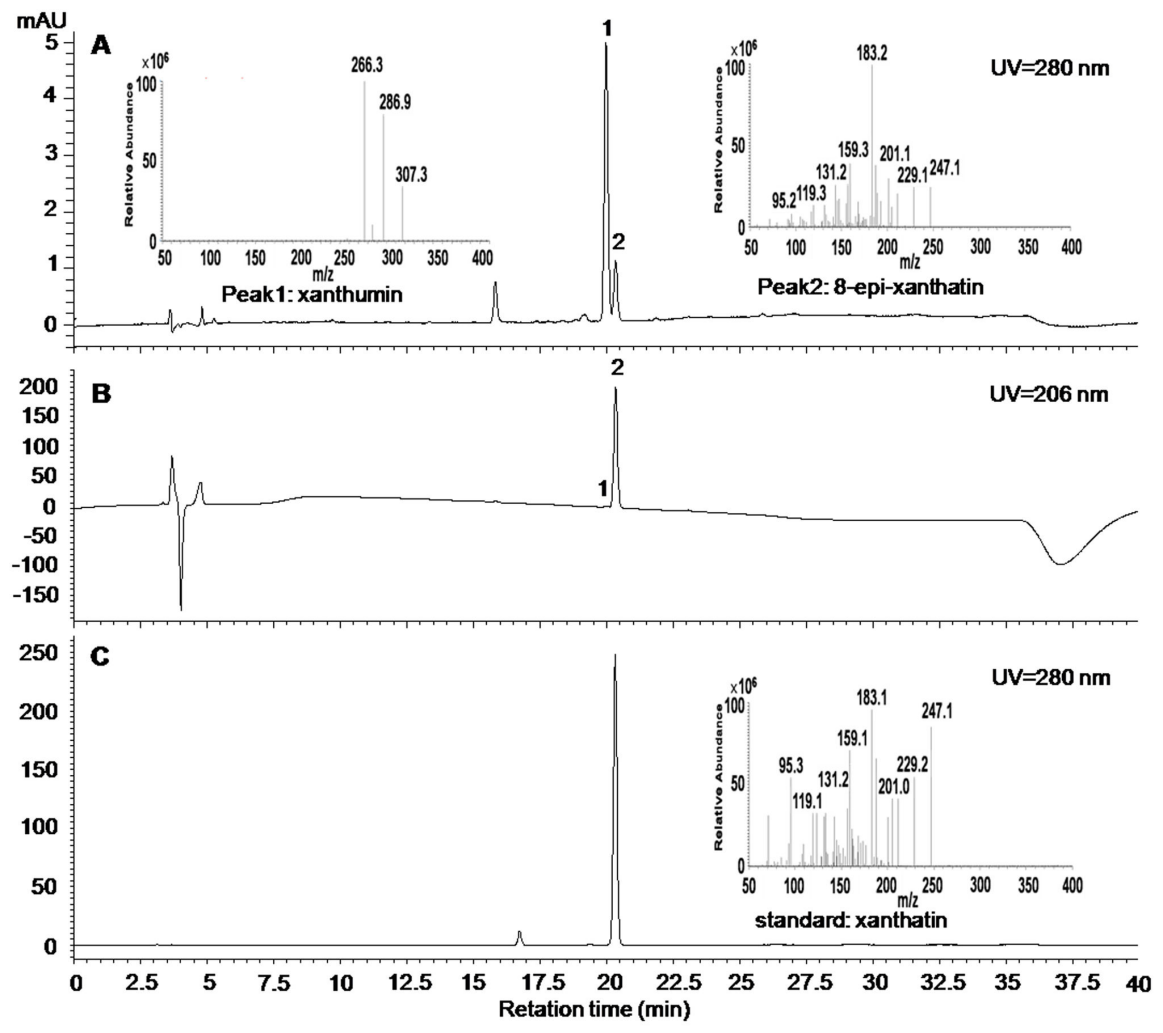
Reversed-phase HPLC profiles of the young leaves extracts of Hubei-Wuhan *X. strumarium* species. LC chromatograms are shown for the extracts at UV wavelengths of 280 nm (A); 206 nm (B), and for the xanthatin standard at 280 nm of UV wavelength (C). Insets: MS spectrums of Peak1, Peak2, and xanthatin standard (the MS spectrums were obtained by LC-MS analysis. Peaks 1 and 2 were assigned as xanthumin and 8-epi-xanthatin respectively by NMR analysis, see the supporting information).

To investigate which organs of *X. strumarium* were actively synthesising the xanthanolide, the plant species from Hubei-Wuhan was used. Several different tissues including roots, stems, young leaves, mature leaves, flowers, and seeds were extracted with chloroform, and the chloroform extracts were air-dried and dissolved in methanol for HPLC and (+) LC-ESI-MS analysis. As shown in the HPLC profile at 280 nm wavelength (Figure 2A), a big peak (designated Peak 1) followed by a small one (designated Peak 2) was observed from the extracts, and eluted at the retention times near to that of xanthatin standard (Figure 2C). The UV-absorption spectra of both Peaks 1 and 2 were divergent, which can be seen by distinct UV responses of the two peaks at 206 nm (Figure 2B). Similar to xanthatin standard, Peak1 (Peak 1 was assigned to xanthumin by the following NMR analysis in this study) had a strong UV absorption at around 280 nm, whereas Peak2 (Peak 2 was assigned to 8-epi-xanthatin by the following NMR analysis in this study) showed a maximum UV absorption at less than 210 nm (Figure S2). Surprisingly, there were no peaks showing the exact same retention times matching the xanthatin standard (Figure 2C). When the same extracts were subjected to LC-ESI-MS analysis, Peaks 1 and 2 showed molecular ions at 307.3 and 247.3, respectively (Figure 2A). The molecular ion represented by the peak at 247.3 in (+)-LC-ESI-MS suggested that the molecular mass of the compound was 246, which was consistent with the molecular formula of xanthatin. Peak2 had similar MS fragmentation products but eluted only after a slightly longer retention times, compared to the xanthatin standard (Figure 2B and C), indicating that Peak2 could be xanthatin isomer. To elucidate its structure, the mixture of both peaks above was collected (since both peaks could not be separated under our preparative HPLC conditions) and subjected to the standard one- and two-dimensional NMR analysis. By comparing our 2D NMR and MS data with the data from xanthatin standard and published literature [11,24,25], Peaks 1 and 2 were assigned to xanthumin and 8-epi-xanthatin, respectively. The major MS fragmentation product of xanthumin, m/z^+^ 266 ion, could be derived from the loss of the acetyl group from the parental ion [M+H]^+^ at m/z 307. NMR results for xanthumin, 8-epi-xanthatin, and xanthatin were all tabulated in Table S1 in the supporting information. Using 8-epi-xanthatin as the example, the procedures of NMR assignments were shown in the supporting information (Figures S3-S7).

**Figure 3:**
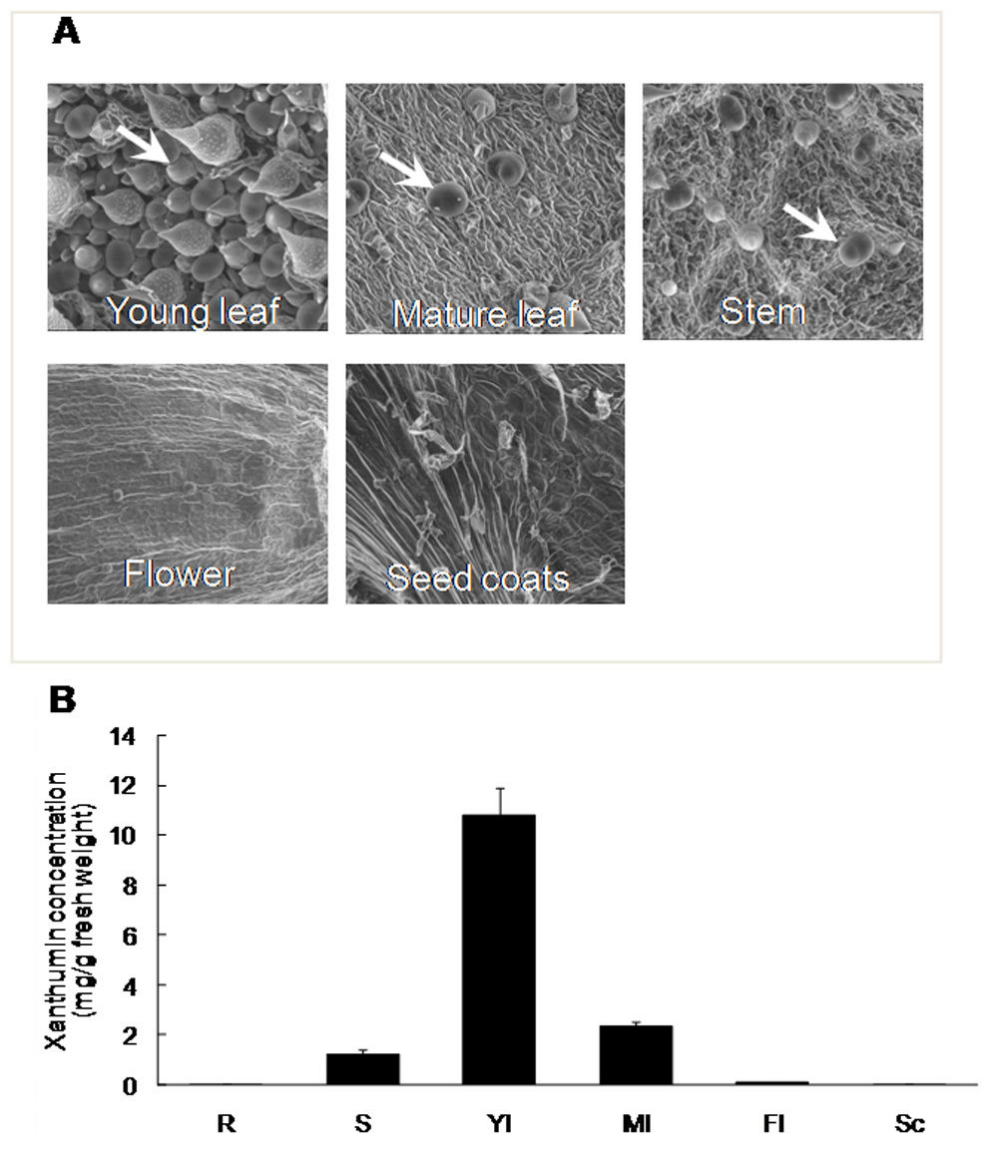
The distribution of glandular trichomes (A) and the biosynthesis of xanthumin (B) in different organs. The *X. strumarium* species from Hubei-Wuhan was used for this experiment (abbreviations: R, roots; S, stems; Yl, young leaves; Ml, mature leaves; Fl, flowers; Sc, seed coats). The glandular trichomes were indicated by white arrows; the error bars represent standard errors from three biological replicates.

Due to the absence of authentic xanthumin, the relative abundance of xanthumin in different tissues was calculated based on the standard xanthatin curves. As shown in Figure 3B, the highest concentration of xanthumin was detected in the young leaves with lower accumulation in the mature leaves, stems, and flowers, while no xanthumin was found in the roots. Similar results were also observed for 8-epi-xanthatin (data not shown). A variety of sesquiterpenoids, such as the anti-malarial compound artemisinin and the anti-tumour compound parthenolide, were reported to be biosynthesized in the glandular trichomes [26,27]. We hypothesized that xanthanolide could also be accumulated in a similar location, the glandular trichomes of *X. strumarium*. To evaluate this possibility, the glandular trichome distribution on the surfaces of the different tissues (young leaves, mature leaves, stems, flowers, and seed coats) was investigated using an SEM. As expected from the chemical data, the highest glandular trichome density was observed on the surface of the young leaves with a lower density on the mature leaves and stems: no trichomes were found on the flowers or seed coats (Figure 3A). The spatial distribution of the glandular cells matched the accumulation of xanthanolides in the different tissues of *X. strumarium*, which strongly suggested that xanthanolides, like other sesquiterpenes, were produced in the glandular trichomes. Isolated glandular trichomes are valuable resources for investigating the key genes involved in terpenoid metabolism, such as monoterpenes and sesquiterpenes, it will be of particular interest to study xanthanolide biosynthesis using the isolated glandular trichomes of *X. strumarium* as the material.

### Chloroform-dipped extracts truly reflected the chemical characteristics inside glandular trichomes

**Figure 4:**
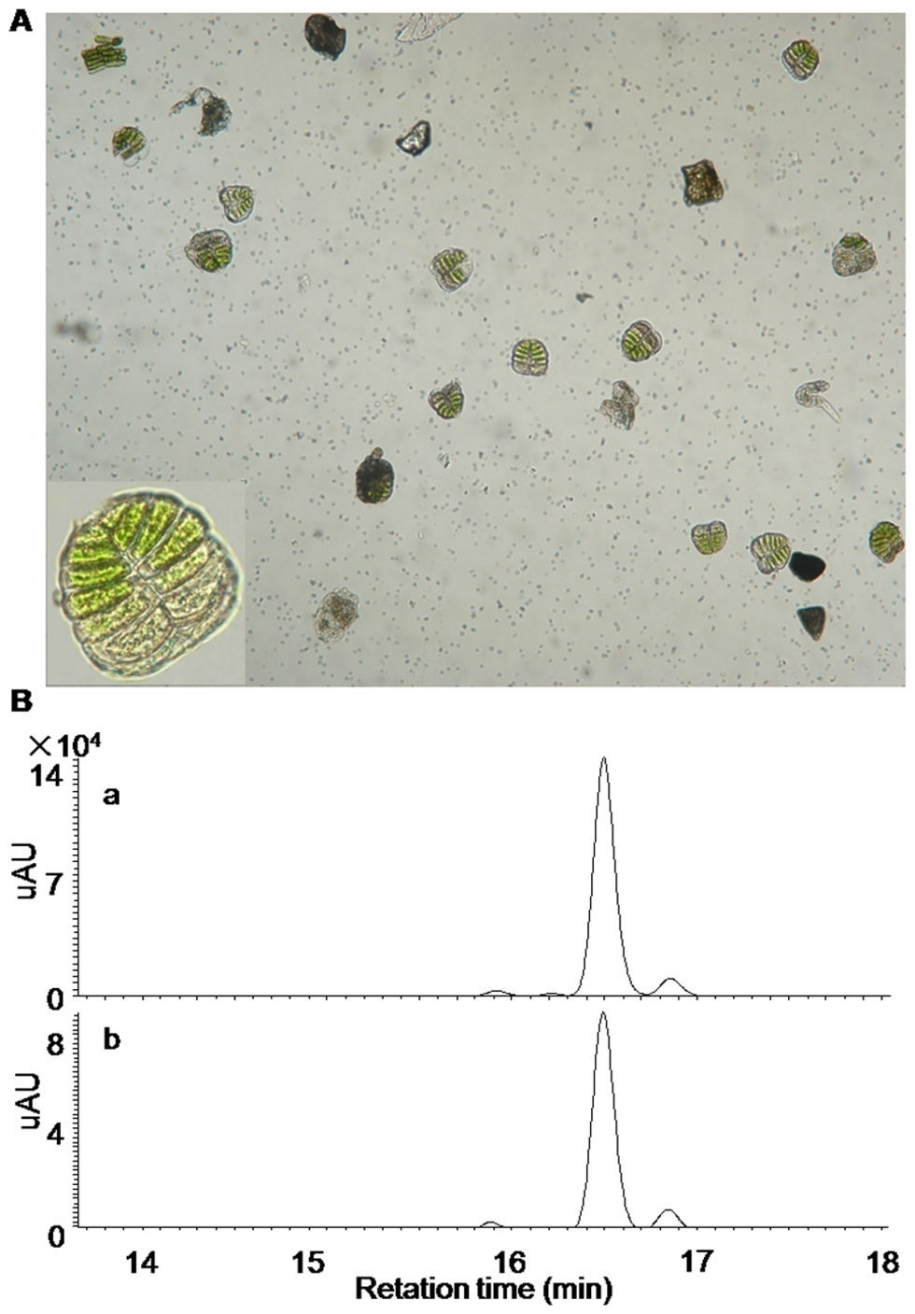
LC-MS analysis of the extracts from the isolated glandular trichomes of *X. strumarium*. (A) The glandular cells purified from Hubei-Wuhan *X. strumarium* species (inset: a single glandular structure at a magnified visual field); (B) the comparison of the LC-MS profiles derived from the chloroform-dipping extracts (a) and the extracts directed from the isolated glandular trichomes (b).

Without isolating glandular trichomes, a chloroform dipping technique has been applied to efficiently extract the chemicals from the glandular cells, but not, or at least much less so, from the remaining plant tissues [28,29]. However, to the best of our knowledge, comparison of the chemical profiles between those chloroform-dipped extracts and those directly extracted from isolated glandular trichomes has never been done. To investigate whether or not those extracts by the chloroform dipping technique truly reflected the chemical characteristics inside the secretory glandular cells, the glandular trichomes were mechanically separated from the young leaves of the Hubei-Wuhan *X. strumarium* species by glass bead-beating, and purified with the gradient sized-filters. As shown in Figure 4A, relatively pure glandular trichomes were obtained and a single *X. strumarium* glandular trichome structure was composed of 6-pairs of cells. Interestingly, although belong to the same Asteraceae family, the glandular cells from *X. strumarium* consists of 6-pairs of cells, whereas the glandular structure from the Chinese anti-malarial plant *A. annua* is organised by 5-pairs of cells [16]. The unique mechanism controlling the development of the glandular cells might exist in *X. strumarium* species. The isolated glandular trichomes were grounded in liquid nitrogen, extracted with chloroform, air-dried, and dissolved in methanol for (+)-LC-ESI-MS analysis. The results showed that the LC-MS profiles from both the procedures (mechanical versus chloroform dipping procedures) were qualitatively similar, which suggested that the chloroform-dipped extracts truly represented the chemical properties of the glandular trichomes (Figure 4B).

### Identify three distinct chemotypes of *X. strumarium* glandular trichomes

**Figure 5:**
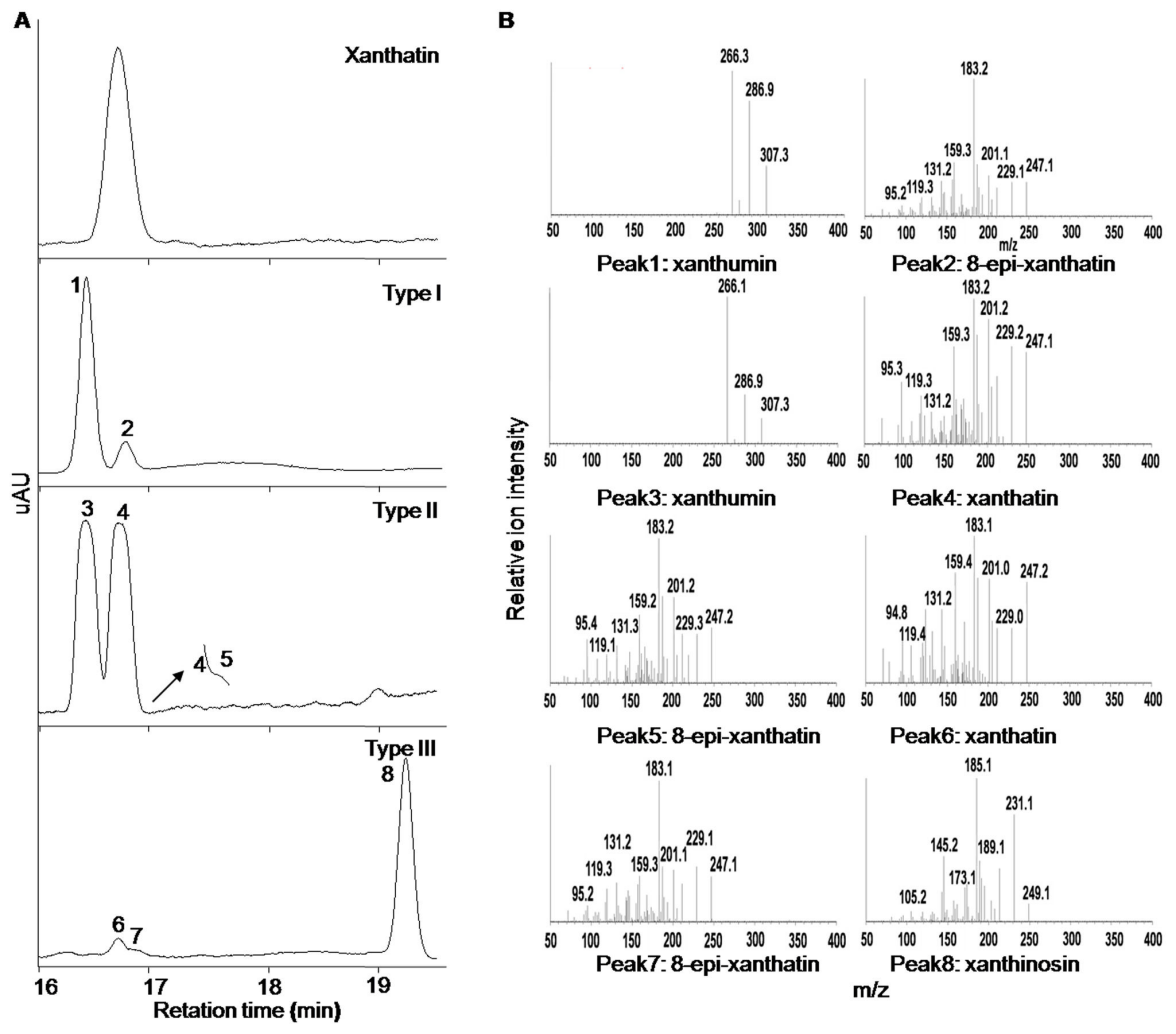
LC-MS analysis of the extracts from three chemotypes of *X. strumarium* glandular trichomes. (A) The representative LC chromatograms at UV wavelength of 280 nm for xanthatin standard, and the glandular extracts from the Type I, Type II, and Type III cells; (B) the mass spectra of xanthatin standard, with each peak labelled. The collision energies used for xanthatin (standard), xanthatin (sample), 8-epi-xanthatin (sample), xanthumin (sample) and xanthinosin (sample) were 15 V, 15 V, 15 V, 13 V and 5 V respectively.

It is well known that biotic and abiotic factors could reprogram the secondary metabolism in plants regulating specific natural product biosynthesis quantitatively or qualitatively. The glandular trichomes mounted on the surfaces of the *X. strumarium*, as the frontier structure interacted with environmental signals, might synthesize the diversified chemicals to protect the plant itself. To investigate the chemical diversity inside the glandular trichomes of the *X. strumarium* species originating from different ecological regions, the seeds of the plant species from the provinces of Hubei (Xianning, Wuhan, Xishui and Fangxian), Anhui (Langxi and Hefei), Hunan (Huaihua), Jiangxi (Ganzhou), Zhejiang (Lishui), Sichuan (Suining, Guanyuan and Qingcheng), Gansu (Qiangyang), Henan (Sanmenxia and Nanyang), Guizhou (Wangmo and Zunyi), Shandong (Taian), and Beijing (Figure S1) were collected and cultivated in the greenhouse of the Wuhan Botanical Garden, Chinese Academy of Sciences. The young leaves of the seedlings obtained were dipped in chloroform for 30 s, and the extracts were subjected to (+)-LC-ESI-MS analysis. Qualitatively, three chemotypes of *X. strumarium* trichomes were observed and designated as Type I, Type II, and Type III cells, respectively (Figure 5A). Of the plant species that we collected, most of them including the species from Hubei-Xianning, Hubei-Wuhan, Hubei-Xishui, Hubei-Fangxian, Henan-Nanyang, Anhui-Langxi, Anhui-Hefei, Hunan-Huaihua, Jiangxi-Ganzhou, Zhejiang-Lishui, Sichuan-Suining, Sichuan-Guangyuan, Sichuan-Qingcheng Guizhou-Wangmo, Shandong-Taian, and Beijing, bore the same chemotype of glandular trichomes that were classed as Type I cells (Table 1). Peaks 1 and 2 in the LC-MS profile of these Type I cells (Figure 5A) have been determined to be xanthiumin and 8-epi-xanthatin as described above (Figure 2A). The glandular trichomes of the plant species from Gansu-Qiangyang and Henan-Sanmenxia were grouped into the Type II chemotype (Table 1). The HPLC profile from the Type II cells is very different from that for Type I. As shown in Figure 5A, two major peaks (Peaks 3 and 4) were observed in the LC-MS profile from the Type II cells, in which Peak3 overlapped Peak4 while a closer observation showed that Peak4 was tailed by another peak (Peak5). By trying different analysis conditions, we failed to separate Peak5 from Peak4. Judged by MS fragmentation products and retention times in HPLC separations (Figure 5A and B), we suspected that Peaks 3, 4, and 5 could be xanthumin, xanthatin, and 8-epi-xanthatin, respectively. Due to the inability to separate these peaks under our preparative HPLC conditions, a mixture of them was collected and analysed by NMR. The NMR results indeed showed the signals for xanthumin, xanthatin, and 8-epi-xanthatin in the Type II cells (see the supporting information). Interestingly, compared to that of Type I or Type II cells, a very different LC-MS profile was observed for the glandular extracts of the Guizhou-Zunyi species (Figure 5A) that was consequently designated as the Type III cells (Table 1). The LC-MS profile showed that three peaks (Peaks 6, 7, and 8) were observed in the Type III chemotype of the species under the analysis conditions (Figure 5A), by comparisons of the MS fragmentation products and HPLC retention times (Figures 5A and 4B), we suggested that Peaks 6 and 7 could be xanthatin and 8-epi-xanthatin respectively. Peak8 had a molecular ion with a mass-to-charge ratio (*m/z* ^*+*^) of 249.2 (Figure 5B), compared to xanthatin molecular ion of *m*/*z* ^+^ = 247.3, indicating that the compound could be double bond saturated xanthatin. When the mixture of them was HPLC collected and subjected to NMR analysis, NMR results confirmed the occurrence of xanthatin, 8-epi-xanthatin, and xanthinosin in this chemotype. NMR signals for each compound were shown in the supporting information. The combination of MS and NMR data strongly suggested that Peaks 6, 7, and 8 in the Type III chemotype were xanthatin, 8-epi-xanthatin, and xanthinosin, respectively.

**Table 1:** The geographic locations of the *X. strumarium* species used in this study and the chemotypes of their glandular trichomes characterized.

Geographic locations		Types	Major xanthanolides
Guizhou	Zunyi	Type III	8-epi-xanthatin, xanthinosin, xanthatin
	Wangmo	Type I	8-epi-xanthatin, xanthumin
Beijing		Type I	8-epi-xanthatin, xanthumin
Shandong	Taian	Type I	8-epi-xanthatin, xanthumin
Anhui	Langxi	Type I	8-epi-xanthatin, xanthumin
	Hefei	Type I	8-epi-xanthatin, xanthumin
Zhejiang	Lishui	Type I	8-epi-xanthatin, xanthumin
Jiangxi	Gongzhou	Type I	8-epi-xanthatin, xanthumin
Hunan	Huaihua	Type I	8-epi-xanthatin, xanthumin
Sichuan	Guanyuan	Type I	8-epi-xanthatin, xanthumin
	Qingcheng	Type I	8-epi-xanthatin, xanthumin
	Suining	Type I	8-epi-xanthatin, xanthumin
Hubei	Wuhan	Type I	8-epi-xanthatin, xanthumin
	Xianning	Type I	8-epi-xanthatin, xanthumin
	Xishui	Type I	8-epi-xanthatin, xanthumin
	Fangxian	Type I	8-epi-xanthatin, xanthumin
Henan	Nanyang	Type I	8-epi-xanthatin, xanthumin
	Sanmenxia	Type II	8-epi-xanthatin, xanthumin, xanthatin
Gansu	Qiangyang	Type II	8-epi-xanthatin, xanthumin, xanthatin

**Figure 6:**
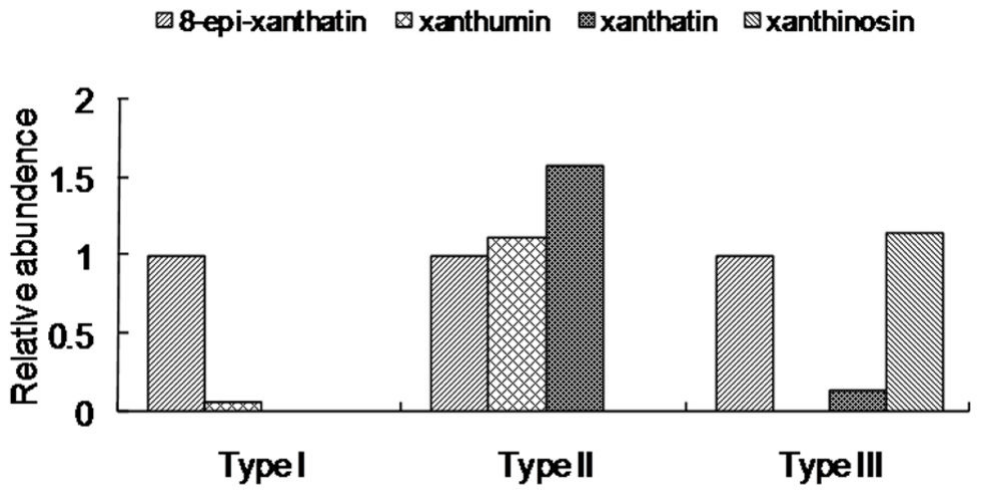
The relative abundance of the xanthanolides in three chemotypes of *X. strumarium* glandular cells. *X. strumarium* trichome extracts from Hubei-Wuhan, Henan-Sanmenxia, and Guizhou-Zunyi were used as the representative extracts for Type I, II, and III glandular cells, respectively. In each chemotype of the glandular cells, the relative abundances of the metabolites were evaluated from the integrals of selected metabolite NMR signals (least overlapping signals) relative to that of 8-epi-xanthatin (We set it as 1) and the accumulation levels of the other xanthanolides were normalized thereto.

The intensities of non-overlapping NMR signals were proportional to the concentrations of specific metabolites and this information was used for the quantification of individual compounds from a sample mixture in a single run. On this aspect, due to the differences in UV absorption and ionisation of metabolites, LC-MS was not available for the comparisons of the concentrations between different metabolites by a single injection. Using deuterated methanol-d4 as the internal reference chemical, relative quantities of the major compounds in each chemotype of *X. strumarium* species were compared by checking specific NMR signals. As shown in Figure 6, the Type I species contained a significantly higher concentration of 8-epi-xanthatin than that of xanthumin, no xanthatin or xanthinosin were detected in this species by NMR and LC-MS analysis. In the Type II species, xanthatin, 8-epi-xanthatin, and xanthumin were detected while no xanthinosin was found, the relative abundance of xanthatin, 8-epi-xanthatin, and xanthumin were comparable. For Type III, 8-epi-xanthatin and xanthinosin were the major chemicals whereas a comparatively lower level of xanthatin was also detected. No xanthumin was observed in this species, interestingly, xanthinosin was only detected in the Type III species indicating a unique ecological importance for this type of *X. strumarium*.

It is not clear which environmental elements are responsible for the differences between the chemotypes. Since the Type III chemotype species were widely distributed from northern to southern areas of China (see Figure S1), it was thought that abiotic factors such temperature and rainfall were unlikely to have caused the differences between chemotypes. Type II and Type III species might be evolved from the Type I chemotype under specific biological pressures such as the stresses from pests or pathogens. There are few phenotypic differences between the chemotypes, the only visible difference being that more purple pigment accumulates at the leaf petioles of Type I plant species than was so for Type II or III species, this would indicate that more flavonoids such as anthocyanin were biosynthesized in the Type I species [30].

### Concluding remarks

For the first time, a unique glandular structure composed of 6-pairs of cells was identified from *X. strumarium*. Our phytochemical studies indicated that xanthanolides (sesquiterpene lactones) of *X. strumarium* were primarily accumulated in the glandular cells. When the glandular secretions were analyzed by NMR and LC-MS analysis, three distinct chemotypes of *X. strumarium* glandular trichomes were discovered by screening the plant species collected from eleven Chinese provinces. The relative abundance of the major chemicals in each chemotype was compared. The question arose as to the different ecological importance of the three chemotypes of *X. strumarium* in their native regions, and the evolutional genetic drivers for the chemical variations in their natural selection.

## Supporting Information

Figure S1 **Locations for the seed samples acquired in this study.** 1, Guizhou -Zunyi; 2, Guizhou -Wangmo; 3, Beijing; 4, Shandong-Taian; 5, Anhui-Langxi; 6, Anhui-Hefei; 7, Zhejiang-Lishui; 8, Jiangxi-Gongzhou; 9, Hunan-Huaihua; 10, Sichuan-Guangyuan; 11, Sichuan-Qingcheng; 12 Sichuan- Suining; 13, Hubei-Wuhan; 14, Hubei-Xianning; 15, Hubei-Xishui; 16, Hubei-Fangxian; 17, Henan -Nanyang; 18, Henan-Sanmenxia; 19, Gansu-Qingyang. ○ (red) indicates Type I species, ○ (blue) indicates Type II species, ○ (green) indicates Type III species.
(TIF)

Figure S2 **UV spectra of xanthatin, 8-epi-xanthatin, xanthumin, and xanthinosin.**
(TIF)

Figure S3 **The ^1^H-^1^H TOCSY spectrum for 8-epi-xanthatin assignment.**
(TIF)

Figure S4 **The ^1^H-^1^H COSY spectrum for 8-epi-xanthatin assignment.**
(TIF)

Figure S5 **The ^1^H-^1^H JRES spectrum for 8-epi-xanthatin assignment.**
(TIF)

Figure S6 **The ^1^H-^13^C HSQC spectrum for 8-epi-xanthatin assignment.**
(TIF)

Figure S7 **The ^1^H-^13^C HMBC spectrum for 8-epi-xanthatin assignment.**
(TIF)

Table S1 **NMR assignments of the major metabolites for xanthatin, 8-epi-xanthatin, xanthinosin and xanthumin in *X. strumarium* glandular cells extracts^a^.**
(DOC)
