# Supplementary figures and images for: Identifying Three Ecological Chemotypes of Xanthium strumarium Glandular Trichomes Using a Combined NMR and LC-MS Method
Source: PLoS One. 2013 Oct 2;8(10):e76621. doi: 10.1371/journal.pone.0076621 (PMC3788720; doi:10.1371/journal.pone.0076621)

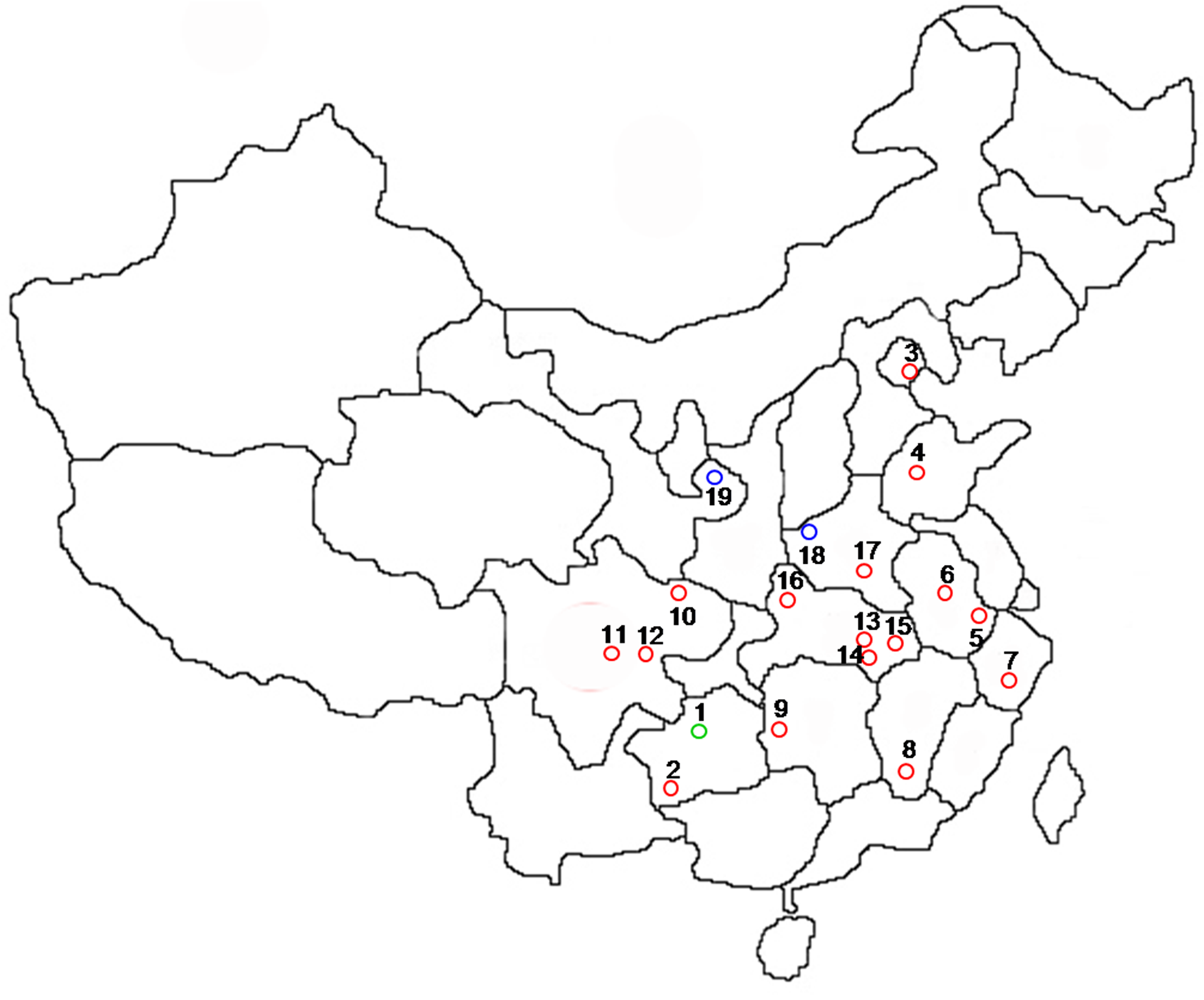

Supplement: Figure S1 — Locations for the seed samples acquired in this study. 1, Guizhou -Zunyi; 2, Guizhou -Wangmo; 3, Beijing; 4, Shandong-Taian; 5, Anhui-Langxi; 6, Anhui-Hefei; 7, Zhejiang-Lishui; 8, Jiangxi-Gongzhou; 9, Hunan-Huaihua; 10, Sichuan-Guangyuan; 11, Sichuan-Qingcheng; 12 Sichuan- Suining; 13, Hubei-Wuhan; 14, Hubei-Xianning; 15, Hubei-Xishui; 16, Hubei-Fangxian; 17, Henan -Nanyang; 18, Henan-Sanmenxia; 19, Gansu-Qingyang. ○ (red) indicates Type I species, ○ (blue) indicates Type II species, ○ (green) indicates Type III species. (TIF) [file pone.0076621.s001.tif]

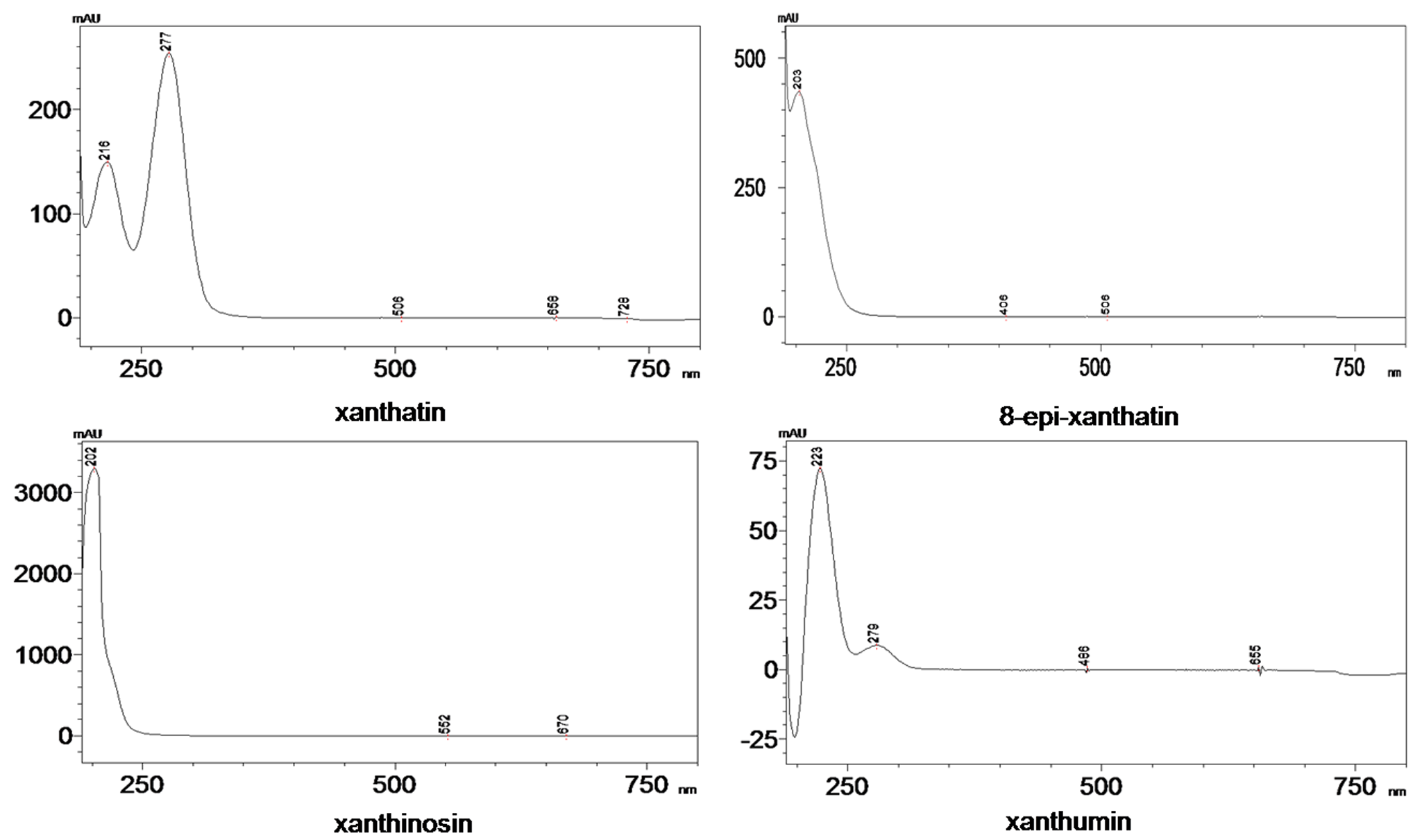

Supplement: Figure S2 — UV spectra of xanthatin, 8-epi-xanthatin, xanthumin, and xanthinosin. (TIF) [file pone.0076621.s002.tif]

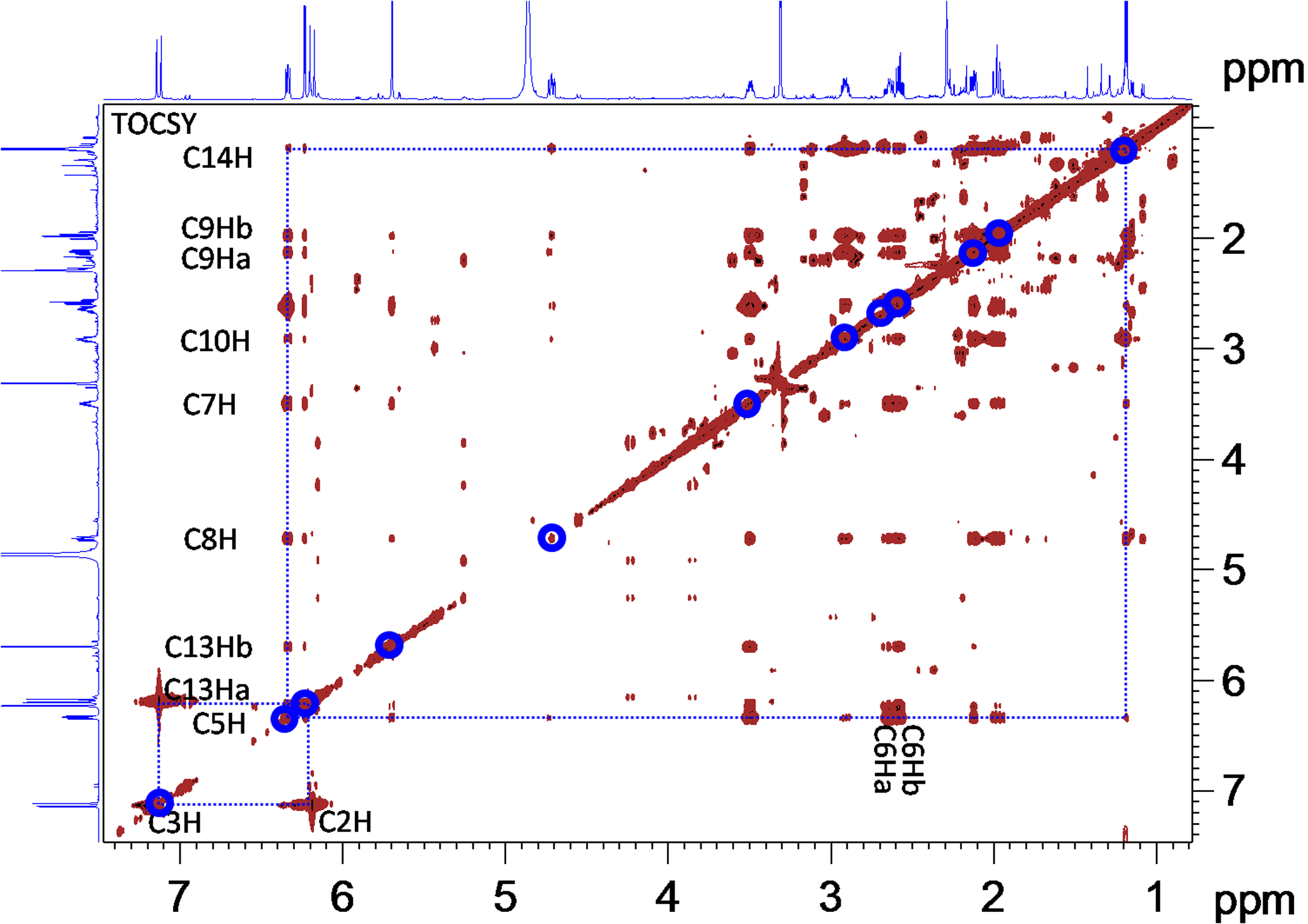

Supplement: Figure S3 — The 1H-1H TOCSY spectrum for 8-epi-xanthatin assignment. (TIF) [file pone.0076621.s003.tif]

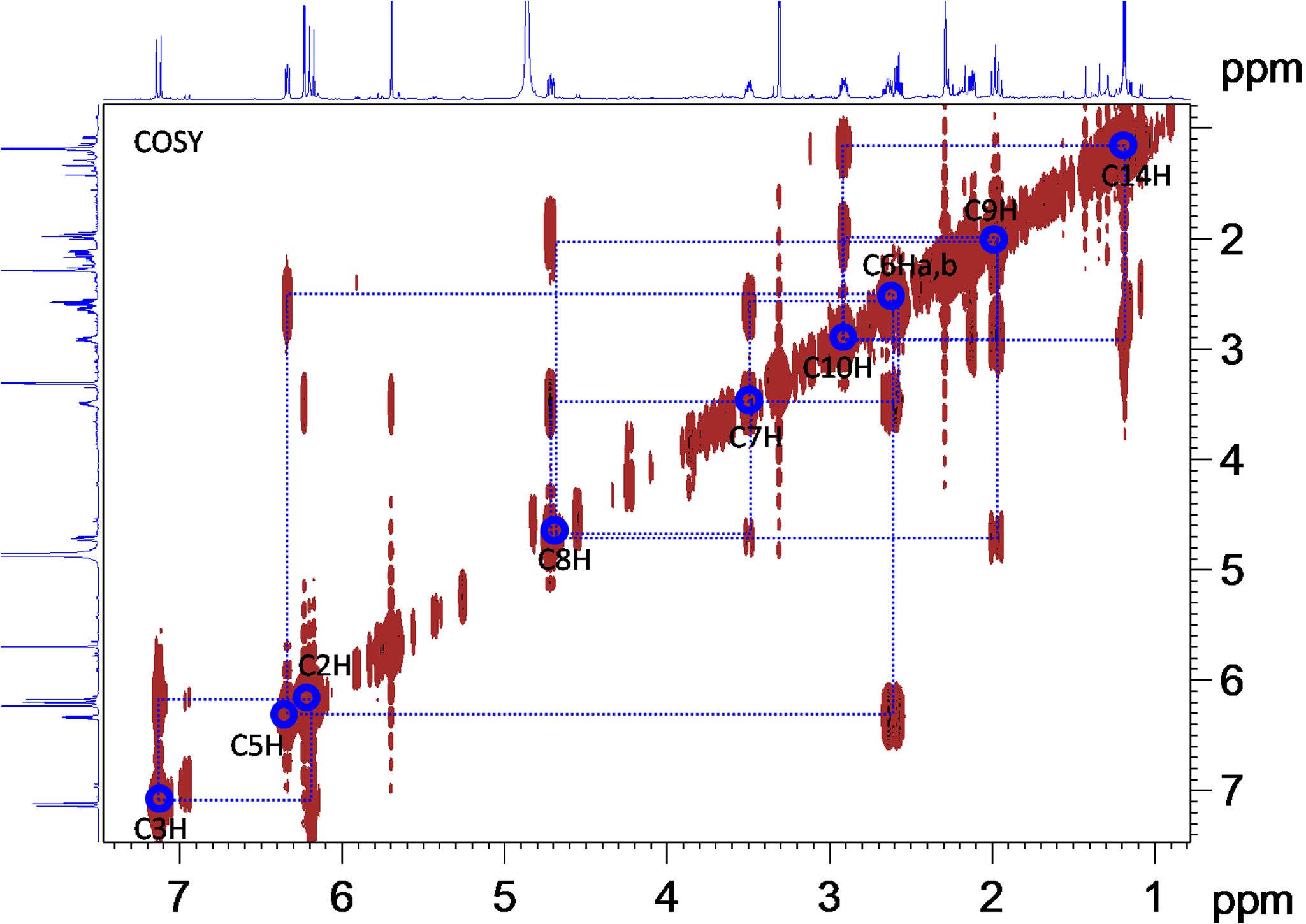

Supplement: Figure S4 — The 1H-1H COSY spectrum for 8-epi-xanthatin assignment. (TIF) [file pone.0076621.s004.tif]

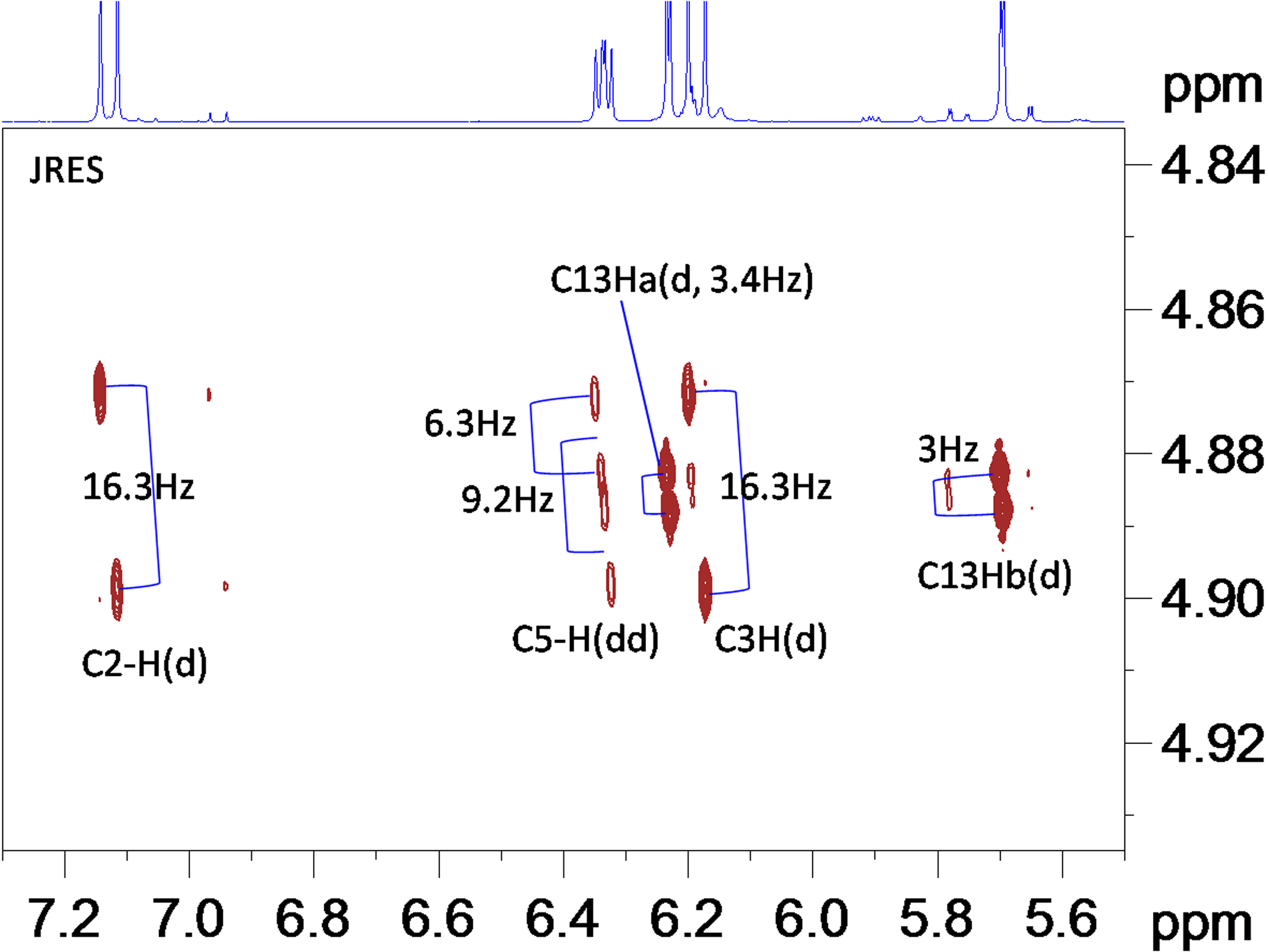

Supplement: Figure S5 — The 1H-1H JRES spectrum for 8-epi-xanthatin assignment. (TIF) [file pone.0076621.s005.tif]

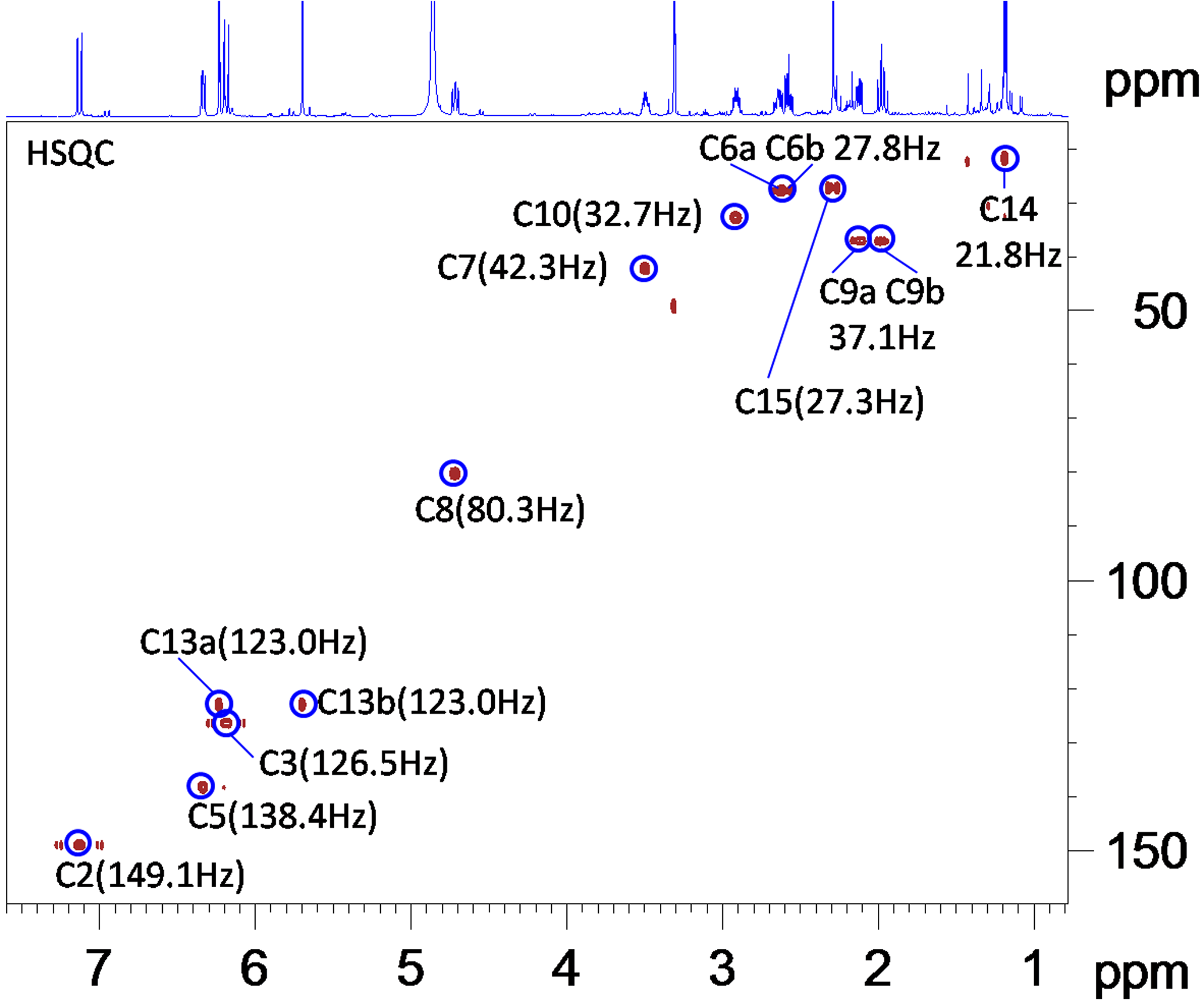

Supplement: Figure S6 — The 1H-13C HSQC spectrum for 8-epi-xanthatin assignment. (TIF) [file pone.0076621.s006.tif]

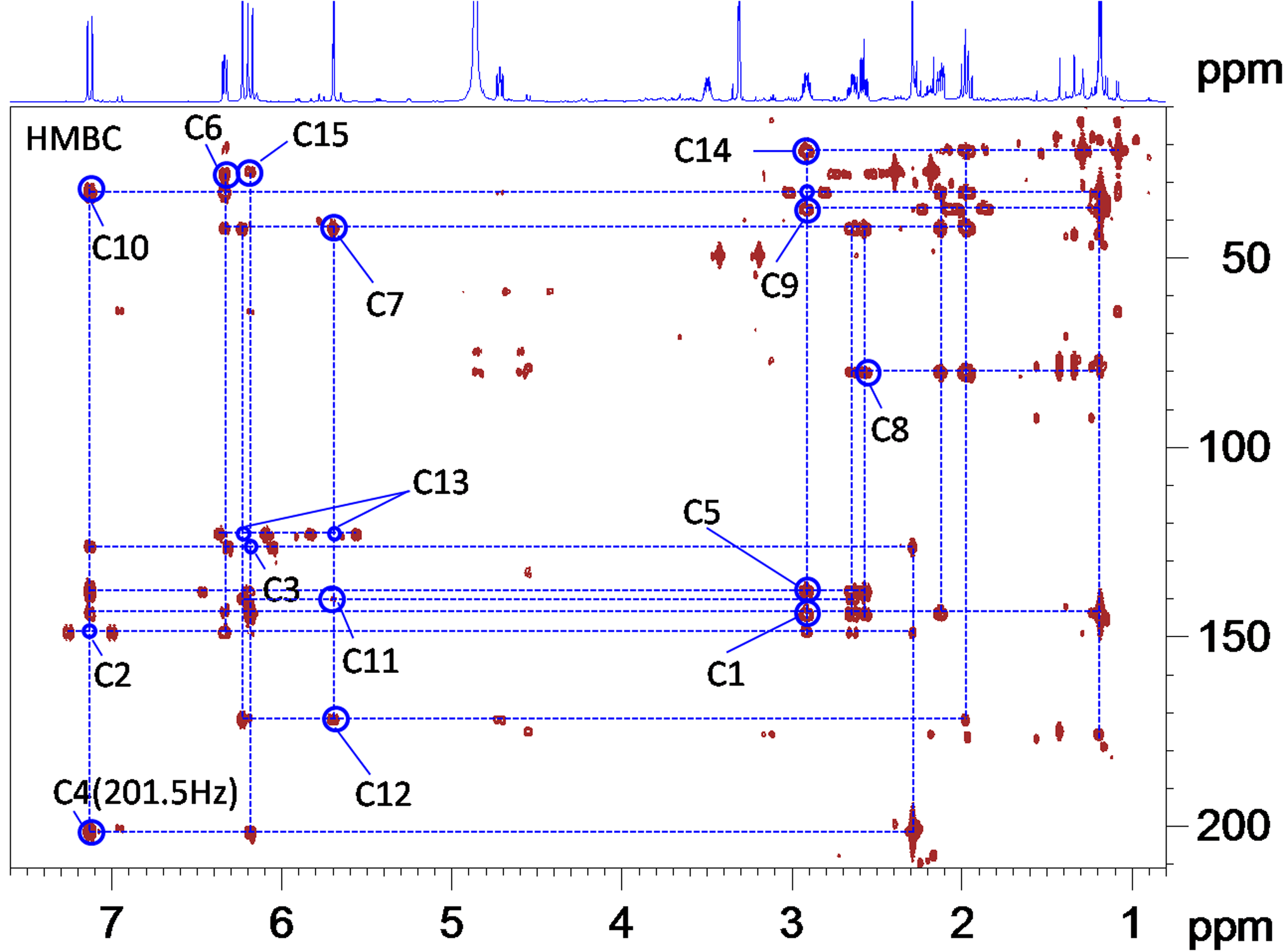

Supplement: Figure S7 — The 1H-13C HMBC spectrum for 8-epi-xanthatin assignment. (TIF) [file pone.0076621.s007.tif]
