# Supplementary material for: Identifying Three Ecological Chemotypes of Xanthium strumarium Glandular Trichomes Using a Combined NMR and LC-MS Method
Source: PLoS One. 2013 Oct 2;8(10):e76621. doi: 10.1371/journal.pone.0076621 (PMC3788720; doi:10.1371/journal.pone.0076621)
Supplement: Table S1 — NMR assignments of the major metabolites for xanthatin, 8-epi-xanthatin, xanthinosin and xanthumin in X. strumarium glandular cells extractsa. (DOC) [file pone.0076621.s008.doc]

Table S1 NMR assignments of the major metabolites for xanthatin, 8-epi-xanthatin, xanthinosin and xanthumin in *X. strumarium* glandular cells extracts^a^.

| No. | Xanthatin |  |  | | 8-epi-Xanthatin |  |  | | Xanthinosin |  |  | Xanthumin |  |
| --- | --- | --- | --- | --- | --- | --- | --- | --- | --- | --- | --- | --- | --- |
|  | *δ*_H_^a^  (298 K) | *δ*_C_ |  | | *δ*_H_^a^ (298 K) | *δ*_C_ |  | | *δ*_H_^a^ (298 K) | *δ*_C_ | | *δ*_H_^a^ (298 K) | *δ*_C_ |
| 1 | - | 144.1 | | - | | 144.1 | | - | | 147.5 | | - | 145.4 |
| 2 | 7.22 (d, 16.1) | 151.1 | | 7.13 (d, 16.3) | | 149.1 | | 2.29 (m) | | 42.0 | | 5.43 (dd, 9.3, 4.0) | 74.5 |
| 3a | 6.27 (d, 16.1) | 125.2 | | 6.19 (d, 16.3) | | 126.5 | | 2.61 (m) | | 43.4 | | 3.00 (dd, 17.1, 4.2) | 48.2 |
| 3b |  |  | |  | |  | |  | |  | | 2.74 (dd, 17.1, 9.3) | 48.2 |
| 4 | - | 201.5 | | - | | 201.5 | | - | | 210.9 | | - | 207.6 |
| 5 | 6.43 (dd, 9.2, 3.3) | 140.9 | | 6.34 (dd, 9.2, 6.3) | | 138.4 | | 5.59 (dd, 8.9, 3.0) | | 122.7 | | 5.91 (dd, 9.0, 5.7) | 125.7 |
| 6a | 2.89 (dddd, 16.7, 9.1, 2.5) | 27.9 | | 2.63 (m) | | 27.8 | | 2.57 (m) | | 27.1 | | 2.46 (m) | 26.9 |
| 6b | 2.29 (m) | 27.9 | | 2.58 (m) | | 27.8 | | 2.05 (m) | | 27.1 | | 2.36 (m) | 26.9 |
| 7 | 2.60 (m) | 48.6 | | 3.50 (m) | | 42.3 | | 2.50 (m) | | 49.4 | | 3.36 (m) | 42.5 |
| 8 | 4.41 (dddd, 12.2, 9.7, 2.6) | 83.3 | | 4.72 (dddd, 12.4, 8.6, 2.4) | | 80.3 | | 4.32 (dddd, 13.2, 8.9, 2.9) | | 83.7 | | 4.67 (dddd, 12.3, 8.6, 2.6) | 80.7 |
| 9a | 2.36 (dddd, 12.9, 4.1, 2.7) | 37.7 | | 2.12 (dddd, 13.9, 6.9, 2.2) | | 37.1 | | 2.25 (dddd, 12.7, 4.2, 3.0) | | 38.1 | | 2.02 (m) | 37.7 |
| 9b | 1.86 (dddd, 13.2, 12.5, 3.9) | 37.7 | | 1.97 (m) | | 37.1 | | 1.79 (dddd, 13.0, 12.3, 3.9) | | 38.1 | | 1.88 (m) | 37.7 |
| 10 | 3.16 (m) | 30.2 | | 2.91 (m) | | 32.7 | | 2.56 (m) | | 35.2 | | 2.67 (m) | 35.2 |
| 11 | - | 141.2 | | - | | 140.0 | | - | | 141.7 | | - | 140.4 |
| 12 | - | 171.8 | | - | | 171.9 | | - | | 172.4 | | - | 172.1 |
| 13a | 6.12 (d, 3.4) | 119.2 | | 6.23 (d, 3.4) | | 123.0 | | 6.08 (d, 3.4) | | 118.5 | | 6.20 (d, 3.3) | 122.8 |
| 13b | 5.61 (d, 3.1) | 119.2 | | 5.70 (d, 3.0) | | 123.0 | | 5.55 (d, 3.2) | | 118.5 | | 5.65 (d, 2.9) | 122.8 |
| 14 | 1.18 (d, 7.5) | 19.2 | | 1.18 (d, 6.9) | | 21.8 | | 1.16 (d, 7.4) | | 18.8 | | 1.15 (d, 6.9) | 21.8 |
| 15 | 2.30 (s) | 27.6 | | 2.29 (s) | | 27.3 | | 2.15 (s) | | 29.9 | | 2.17 (s) | 30.3 |
| OAc |  |  | |  | |  | |  | | O-C=O | | - | 171.9 |
| OAc |  |  | |  | |  | |  | | CH_3_- | | 1.98 (s) | 20.9 |
| ^a^ Multiplicity, *J*, Hz. | | | | | | | | | | | |  |  |
